# MosQNet-SA: Explainable convolutional-attention network for mosquito classification with application as a RESTful API for dengue and malaria risk mapping

**DOI:** 10.1371/journal.pone.0344970

**Published:** 2026-04-08

**Authors:** Md. Akmol Masud, Sanjida Akter, Nadia Sultana, Mohammad Shahidul Islam, Mohammed Abu Yousuf, Farzan M. Noori, Md Zia Uddin

**Affiliations:** 1 Institute of Information Technology, Jahangirnagar University, Savar, Dhaka, Bangladesh; 2 Department of Informatics, University of Oslo, Oslo, Norway; 3 Sustainable Communication Technologies, SINTEF Digital, Oslo, Norway; Newcastle University, UNITED KINGDOM OF GREAT BRITAIN AND NORTHERN IRELAND

## Abstract

Mosquito-borne diseases represent a significant global health challenge. Over 700,000 people succumb to mosquito-borne diseases annually, highlighting the important need for accurate and efficient mosquito classification systems. Current approaches face limitations in accuracy, computational efficiency, and interpretability, creating a gap that artificial intelligence can help address. This paper presents MosQNet-SA, a novel convolutional-attention network designed for mosquito classification that addresses these limitations through architectural choices. The proposed model incorporates a spatial attention mechanism and depthwise separable convolutions to enhance feature extraction while maintaining computational efficiency—achieving comparable performance with 10-fold fewer parameters than existing approaches. MosQNet-SA achieves 99.42% accuracy on a dataset of 1,000 images across three mosquito species (*Aedes*, *Anopheles*, and *Culex*), demonstrating strong performance compared to existing CNN architectures. The model’s explainability is enhanced through multiple methods, including Saliency, GradCAM, LIME, and Kernel SHAP, providing valuable insights into the decision-making process for public health practitioners. Additionally, we present a RESTful API implementation for real-time mosquito classification and disease risk mapping, demonstrating the practical applicability of our approach in public health surveillance systems.

## 1 Introduction

Vector-borne diseases pose a major global health threat, significantly impacting public health systems, especially in tropical and subtropical regions. Diseases spread by mosquito vectors are among the major healthcare challenges worldwide today [1]. Diseases such as dengue fever, malaria, zika, chikungunya, yellow fever, leishmaniasis, and lymphatic filariasis claim millions of lives annually [2]. Mosquitoes are small insects known for their ability to transmit disease to humans and animals through bites, accounting for nearly 70% of global vector-borne diseases [3,4]. Climate change, urbanization, and globalization have further exacerbated the prevalence and geographic spread of these diseases, making control a substantial global challenge.

### 1.1 The global burden of mosquito-borne diseases

Mosquito-borne diseases represent a persistent and severe health challenge. Over 700,000 people succumb to mosquito-borne diseases annually [5], with malaria alone claiming more than 600,000 lives and affecting 250 million people worldwide in 2022 [6]. The World Malaria Report 2023 reveals that climate change is exacerbating this challenge, as variations in temperature, humidity, and precipitation directly affect the behavior and viability of malaria-transmitting Anopheles mosquitoes [7].

Dengue fever presents an equally formidable threat, ranking as the second most lethal mosquito-borne disease with approximately 20,000–21,000 deaths annually worldwide, particularly devastating regions across Asia, the Americas, and Africa [6]. The scale of this epidemic became starkly evident on April 30, 2024, when the WHO reported more than 7.6 million dengue cases globally, including 3.4 million confirmed cases, over 16,000 severe cases, and more than 3,000 deaths [8]. The Aedes mosquito genus, particularly *Aedes aegypti*, serves as the primary vector for dengue, Zika virus, chikungunya, and urban yellow fever, with these mosquitoes typically feeding during daylight hours [9]. Despite available vaccines for yellow fever, the disease continues to cause 30,000 deaths annually worldwide, while lymphatic filariasis burdens approximately 657 million people across 39 countries [10].

### 1.2 Current control strategies and their limitations

Traditional approaches to controlling vector-borne diseases rely on an integrated strategy combining insecticide-treated nets, indoor residual spraying, environmental management, surveillance, and health education. However, these conventional methods face mounting challenges that limit their effectiveness. Vector populations are rapidly adapting to insecticides, creating resistance that undermines control efforts. Additionally, inadequate health infrastructure in endemic regions and the lack of efficient diagnostic tools further complicate disease management [10]. These persistent challenges necessitate innovative, technology-driven solutions that can adapt to changing vector behaviors and environmental conditions.

Recent scientific advances offer promising new avenues for addressing these limitations. Breakthroughs in remote sensing, geographic information systems (GIS), and environmental modeling have significantly improved our capacity to predict disease transmission patterns and implement preemptive measures. However, a substantial gap remains in developing accurate, efficient, and accessible tools for real-time mosquito identification and classification—a prerequisite for effective surveillance and targeted intervention strategies.

### 1.3 AI-driven solutions for mosquito classification

Artificial intelligence methods hold significant potential for revolutionizing mosquito surveillance and vector-borne disease control [11]. The integration of AI and machine learning has transformed vector-borne disease management by enabling more precise identification of disease vectors, mapping potential breeding sites, and optimizing resource allocation. When applied to diagnostic tools, environmental data analysis, and predictive modeling, these technologies offer new opportunities for early disease detection, real-time monitoring, and better-informed public health interventions.

Convolutional neural networks (CNNs) and attention mechanisms have emerged as particularly promising approaches for enhancing disease detection, surveillance, and risk mapping. CNNs excel at image classification tasks, and when combined with attention mechanisms, they can focus on the most relevant features of the input data, thereby boosting both accuracy and interpretability in disease classification. This capability becomes essential when dealing with the subtle morphological differences between mosquito species that are often indistinguishable to the human eye but carry vastly different disease transmission risks.

To address the pressing challenge of vector-borne disease classification, this study introduces a novel deep-learning model that integrates CNNs with attention mechanisms to classify mosquito species with high accuracy and efficiency. The proposed model incorporates explainable AI techniques and the RESTful API for easy integration into applications for real-time mosquito classification and dynamic disease risk mapping. This research presents the following key contributions:

- Development of MosQNet-SA: A novel deep learning architecture designed to classify mosquito species while reducing computational complexity and inference time, making it suitable for deployment on edge devices.
- Explainable AI Techniques: Integrating explainable AI methods such as Grad-CAM and SHAP ensures the model’s decision-making process is interpretable and transparent, enhancing its reliability in real-world applications.
- Real-Time Applications: The model is designed for scalability and real-time usage, with significantly fewer parameters than existing models, enabling dynamic risk mapping and mosquito classification through a RESTful API.

The subsequent sections of this work are organized as follows: section 2 reviews relevant literature and provides necessary background on the topic. section 3 details the methodology used in developing the MosQNet-SA model. section 4 presents experimental results and model evaluations, including performance comparisons with state-of-the-art models. Explainable AI techniques and their role in enhancing the model’s interpretability are demonstrated in section 5. section 6 presents RESTful API integration of the proposed model. section 7 discusses the findings and explores the implications within the broader research context. Finally, the paper concludes in section 8, summarizing key findings and potential future research directions.

## 2 Literature review

The classification of mosquito species has become an essential focus in the global fight against vector-borne diseases. In recent years, a surge of research has applied machine learning and deep learning techniques to automate and improve the accuracy of mosquito identification. These advances can revolutionize disease control efforts by enabling faster, more precise, and more accessible methods of identifying disease-carrying mosquitoes. This section reviews the evolution of mosquito classification approaches from traditional CNN methods to hybrid approaches and explainable AI techniques, and identifies key challenges and opportunities for advancement.

### 2.1 CNN-based classification approaches

Convolutional neural networks have emerged as the dominant approach for image-based mosquito classification, demonstrating high accuracy in distinguishing different species and genera. Park et al. [12] achieved over 97% accuracy in classifying eight mosquito species using fine-tuned Deep CNNs, leveraging a large dataset of 3,600 images capturing various postures and deformations of mosquitoes. This study underscored the potential of transfer learning in this domain by applying data augmentation techniques and fine-tuning pre-trained models. Building on this foundation, researchers [13] proposed an innovative pipeline for low-cost IoT sensors, using models like VGG16, ResNet50, and custom CNNs to achieve 98% accuracy. This work is particularly notable for addressing the vital need for cost-effective, deployable solutions in resource-constrained settings where traditional laboratory-based identification methods are impractical.

Similarly, Asgari et al. [14] modified the VGG16 architecture, achieving accuracy between 94.66% and 98.92% across multiple datasets. The study included extensive ablation experiments to identify optimal architectural modifications, further advancing CNN-based classification. Siddiqui and Jain [15] tackled the challenging task of distinguishing between mosquito genera, developing a CNN model that achieved 84.51% accuracy on a dataset of 1,800 images from three genera (*Aedes*, *Anopheles*, and *Culex*). Though the accuracy was lower than some other studies, this work highlighted the difficulties in classifying visually similar genera. A comparative study by Okayasu et al. [16] found that deep learning methods, especially ResNet, outperformed traditional feature-based methods, but that data augmentation was necessary to achieve this improvement.

### 2.2 Data modalities and feature optimization

Mosquito classification relies on various data types, including morphological, genetic, and image-based data. Mosquito classification using image data leverages advanced computer vision techniques, specifically CNNs, to improve species identification and lifecycle-stage classification. Studies have shown that images of wings provide better classification performance compared to body images, with CNNs achieving up to 99% accuracy in distinguishing Aedes species [17]. This finding is noteworthy as wing morphology contains species-specific characteristics that are less variable than body features across different environmental conditions and specimen preparations.

Researchers have demonstrated that focusing on prominent anatomical features can significantly improve classification accuracy. Studies using deep CNNs to classify mosquito photos have achieved 94% accuracy by focusing on distinctive features, such as white band stripes on the legs and thorax [18]. Kumar et al. [19] further validated the effectiveness of deep learning models, specifically DCNNs and pre-trained models, in accurately distinguishing mosquito species from image data through hyperparameter optimization and data augmentation, achieving enhanced precision and F1 scores.

### 2.3 Hybrid and multimodal approaches

As the field matures, hybrid and novel approaches have emerged, integrating multiple data types and new methodologies to overcome the limitations of single-modality approaches. De Lima et al. [20] integrated AI with wing geometric morphometry, achieving accuracy rates of 84%−95%. This work represents a significant advancement in combining traditional morphometric analysis with machine learning for mosquito classification, demonstrating how domain expertise can enhance automated approaches.

Beyond visual data, researchers have explored alternative sensing modalities. Genoud et al. [21] investigated optical signals from mosquitoes and found that Support Vector Machines were the most effective for complex classification tasks. Wei et al. [22] proposed WbNet, a novel ResNet-based model that integrates self-attention and data augmentation methods for classifying mosquito species based on wing-beating sounds. This approach achieved 89.9% accuracy on the WINGBEATS dataset, with 100% precision, recall, and F1 Scores for Aedes and Culex species. These hybrid approaches demonstrate the potential for combining various data modalities—visual, audio, and contextual—to improve classification accuracy and robustness further.

### 2.4 Explainable AI for mosquito classification

Explainable AI (XAI) has emerged as a crucial component for enhancing the interpretability and trustworthiness of machine learning models in mosquito classification. This capability becomes particularly important in public health applications where understanding model decisions is essential for practitioner acceptance and regulatory approval. Numerous studies have used XAI methods to improve classification accuracy while clarifying AI models’ decision-making processes.

Adhane et al. [18] employed Grad-CAM visualizations to develop an AI system capable of elucidating mosquito classification decisions. These visualizations emphasize the image components that influence classification decisions, facilitate error analysis, and enhance comprehension of the model’s predictions. Building on this work, the EfficientNet-B0 model used Grad-CAMs to highlight critical image pixels, focusing on the mosquito’s abdomen to precisely distinguish gonotrophic stages [23]. Goni et al. [24] focused specifically on malaria diagnosis through explainable AI, employing SHAP (SHapley Additive exPlanations) to clarify model decisions and provide insights into feature importance. In [25], researchers developed a formal concept lattice method for explainable AI, enabling mosquito classification by providing both accurate local and global explanations for classification decisions.

### 2.5 Research gaps and future directions

Despite significant progress, several critical challenges persist across the field. Limited dataset sizes and diversity, difficulties distinguishing visually similar species, issues with image quality, generalization across different environments, and the need to balance accuracy with computational efficiency represent prominent challenges identified in the literature. Joshi et al. [26] synthesized findings from 120 papers, offering valuable insights into the field’s current state and outlining potential future directions for machine learning in mosquito control. Rodriguez et al. [27] highlighted the potential of combining machine learning with citizen science initiatives, particularly for classifying disease-carrying mosquitoes using crowdsourced data.

These challenges highlight the need for more robust, efficient, and interpretable models that can operate effectively in real-world conditions while maintaining high accuracy and providing meaningful insights into their decision-making processes.

**Fig 1:**
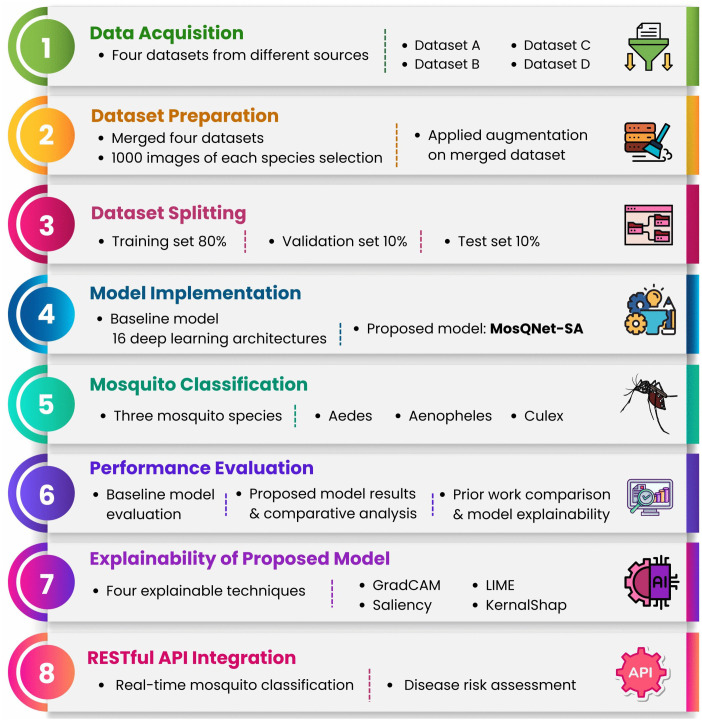
Workflow for mosquito classification and disease risk assessment.

Given these challenges, our novel MosQNet-SA model emerges as a promising solution to advance mosquito classification. The model incorporates many data modalities to enhance robustness. Key domains for continued advancement include multimodal data integration, adaptive learning for continuous model improvement, intuitive interfaces for academics and citizen scientists, and a scalable architecture capable of accommodating new species and geographic regions. Utilizing various explainable AI (XAI) methodologies for mosquito species categorization provides supplementary insights into model decision-making. Multiple explainability methods, such as Saliency, GradCAM, LIME (Local Interpretable Model-agnostic Explanations), and Kernel SHAP (SHapley Additive Explanations) were utilized. Furthermore, including MosQNet-SA into a RESTful API provides a scalable, stateless framework for real-time mosquito categorization and disease risk evaluation. Fig 1 illustrates the comprehensive process used in this research.

## 3 Methodology

This section presents a comprehensive methodology for developing MosQNet-SA, a novel convolutional-attention network for mosquito classification. Our approach addresses the critical challenges identified in the literature review by implementing rigorous data handling procedures, innovative architectural design, and comprehensive evaluation strategies. The methodology is organized into five main components: (1) data collection and preprocessing from multiple sources, (2) data splitting strategy to prevent leakage, (3) statistical validation framework, (4) MosQNet-SA architecture design, and (5) explainable AI implementation. This systematic approach ensures robust, reproducible, and statistically sound results while maintaining computational efficiency and interpretability.

The proposed MosQNet-SA model targets explicitly the balance between accuracy and computational efficiency, incorporating spatial attention mechanisms and depthwise separable convolutions to achieve competitive performance with significantly fewer parameters than existing approaches. The methodology emphasizes explainability through multiple XAI techniques, ensuring that the model’s decisions are transparent and interpretable for public health practitioners.

### 3.1 Dataset acquisition and preparation

**Fig 2:**
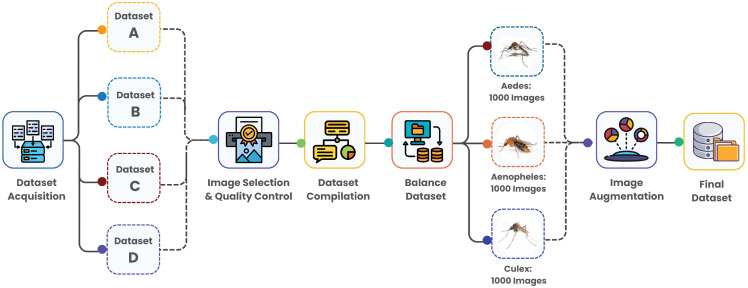
The workflow depicts the creation of a balanced mosquito species dataset. Images were sourced from MosquitoAlert.com, Mendeley Data, IEEE DataPort, and the Dryad Digital Repository, and then selected and quality-controlled to ensure high-quality images. The dataset includes 1000 images each for *Anopheles*, *Aedes*, and *Culex*. Image augmentation techniques enhanced diversity, leading to the final dataset used for model training.

The data set was compiled from four distinct sources to ensure a diverse and comprehensive representation of mosquito species. Fig 2 shows the detailed workflow to create a balanced dataset of mosquito species for this research.

- **Dataset A:** This citizen science platform [28], dedicated to monitoring and controlling mosquito populations, contributed 1,234 images of various mosquito species, including *Anopheles*, *Aedes*, and *Culex*. The photos, submitted by participants worldwide, were meticulously screened and vetted for quality and accuracy.
- **Dataset B:** A publicly accessible dataset from the Mendeley Data repository [29] provided 876 well-annotated images of *Aedes* and *Culex* mosquitoes. The images selected by researchers are particularly well-suited for machine learning tasks.
- **Dataset C:** The IEEE DataPort repository [30], maintained by the Institute of Electrical and Electronics Engineers (IEEE), offered 748 images specifically focused on *Aedes* and *Culex* mosquito species. The research community’s creation of this dataset ensured high relevance and reliability.
- **Dataset D:** Sourced from the Dryad Digital Repository [31], which hosts research data associated with scholarly publications, 600 images of *Anopheles* mosquitoes were included. Extensive metadata and annotations were present in these images.

Images were selected based on quality metrics including resolution (minimum 224x224 pixels), clarity (sharpness score >0.7), and proper species identification verified by entomologists.

#### 3.1.1 Merged dataset.

**Fig 3:**
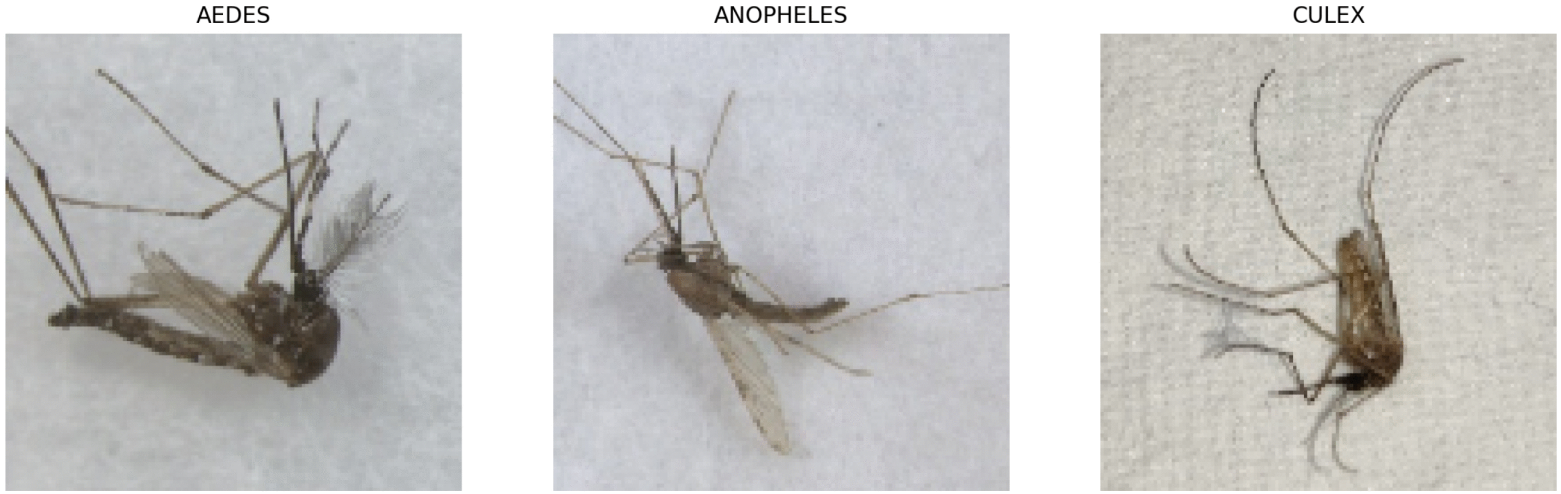
Single instance of *Aedes*, *Anopheles*, and *Culex* mosquito.

The images from these diverse sources were carefully inspected and selected based on the quality, clarity, and suitability for the research objectives of this study. The research dataset [32] includes three significant mosquito species: *Anopheles*, *Aedes*, and *Culex*, each represented by 1,000 images. Fig 3 represents the single instances of the three species of mosquitoes. These species were selected for their significant role in transmitting various mosquito-borne diseases and for their prevalence across different regions worldwide. Ensuring an equal distribution of 1,000 images per species was a deliberate choice to maintain a balanced representation and prevent potential biases during model training and evaluation (Table 1).

**Table 1:** Distribution of images across data sources in train/validation/test splits.

Source	Train	Val	Test	Total
Dataset A	267	33	34	334
Dataset B	266	33	34	333
Dataset C	266	34	33	333
Dataset D	266	33	34	333
Total per species	800	100	100	1000

### 3.2 Augmented merged dataset

Multiple image augmentation techniques were applied to enhance the dataset’s diversity and robustness. These augmentation techniques were used to introduce variability and improve the generalization capabilities of the machine learning models. Table 2 demonstrates the summary of applied augmentation techniques.

**Table 2:** The augmentation techniques that were applied to the merged dataset.

Augmentation	Description
Width Shift Range	Shift the width by up to 30%
Height Shift Range	Shift the height by up to 20%
Shear Range	Apply shear transformation by 10%
Zoom Range	Apply zoom transformation by 40%
Horizontal Flip	Enable horizontal flipping
Fill Mode	Nearest neighbor filling mode

### 3.3 Data splitting strategy

To prevent data leakage and ensure robust evaluation, we implemented a stratified splitting strategy that respects data source boundaries. The dataset was divided into training (80%), validation (10%), and test (10%) sets using the following methodology:

- Per-source stratification: Each data source (A, B, C, D) was split independently before merging, ensuring no cross-source contamination between training and test sets.
- Near-duplicate removal: We applied perceptual hashing (pHash) with a similarity threshold of 0.95 to detect and remove near-duplicate images across all splits. A total of 47 near-duplicates were identified and removed.
- Species and pose stratification: Within each source split, images were balanced across species and pose variations to maintain representative distributions.
- Background stratification: Images with different background types (natural, laboratory, plain) were proportionally distributed across splits.

### 3.4 Baseline model development

For experiments, a range of deep learning architectures, from classic models to advanced designs, have been explored and chosen for effectiveness in image classification tasks. VGG models, such as VGG16 and VGG19, use small receptive fields and a uniform architecture, though they require substantial computational power. ResNet, with its residual connections, solves the vanishing gradient problem, enabling deeper networks such as ResNet50 and ResNet101. Inception and its successor, Xception, improve feature extraction via multi-scale convolutions, with Xception additionally using depthwise separable convolutions for greater efficiency. Inception-ResNet combines these strengths by merging Inception modules with residual connections for improved training stability and accuracy.

DenseNet introduces dense connectivity, in which each layer connects to all previous layers, thereby optimizing feature reuse. MobileNet and EfficientNet focus on reducing computational costs. MobileNet employs depthwise separable convolutions for real-time applications on resource-constrained devices, while EfficientNet uses a compound scaling method to balance network depth, width, and resolution. NASNetMobile, built through neural architecture search, optimizes model performance on mobile platforms by reducing computational demand while maintaining high accuracy. Together, these models provide a diverse toolkit for evaluating performance across varying levels of accuracy, computational efficiency, and suitability for real-time and resource-constrained environments.

### 3.5 Baseline model construction and training setup

Sixteen models with diverse architectures were selected to comprehensively evaluate model performance. This selection includes classical architectures like VGG16 and VGG19, along with more advanced models such as ResNet variants (ResNet50, ResNet101, ResNet152) and others like Xception, InceptionV3, and InceptionResNetV2. Efficient models for mobile environments, such as MobileNet, MobileNetV2, and NASNetMobile, were also included. The DenseNet (121, 169, 201) and EfficientNet (B0, B1, B2) families were selected because they employ dense connectivity patterns (where each layer receives inputs from all previous layers) and compound scaling (systematically increasing network depth, width, and resolution), making them particularly effective for complex classification tasks while maintaining computational efficiency.

All baseline models were trained using consistent procedures to ensure fair comparison: identical data augmentation, same train/validation/test splits, consistent learning rate schedules (cosine annealing), and early stopping with patience of 15 epochs based on validation loss. All models were trained with a learning rate of 0.001, with the number of epochs varying between 38 and 121 depending on the convergence.

### 3.6 MosQNet-SA: Proposed classification model

**Fig 4:**
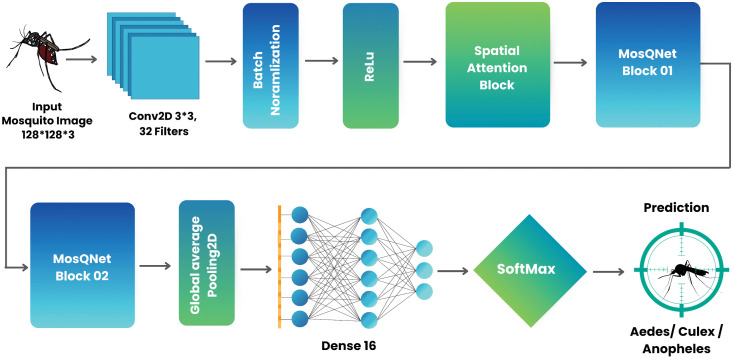
Detailed workflow of MosQNet-SA architecture for classifying mosquito species images using convolutional layers, attention mechanisms, and a novel MosQNet block.

MosQNet-SA is a novel CNN designed specifically for mosquito classification. Its architecture integrates several vital components to enhance feature extraction and improve classification accuracy. In Fig 4, the overall architecture of the MosQNet-SA model is presented for the classification of mosquito species.

The model begins with a convolutional layer for extracting low-level features (edges, textures) and incorporates residual blocks (which allow gradients to flow directly through skip connections), enabling deeper training without vanishing gradient problems. Inception-like blocks perform multi-scale feature extraction (capturing both fine details and broader patterns), while MBConv blocks (mobile-optimized convolutions that separate spatial and channel-wise operations) enhance computational efficiency. The spatial attention block (which learns to focus on the most relevant image regions) allocates computational resources to key areas, improving accuracy in noisy or cluttered environments. The final classification layers aggregate these processed features to make accurate species predictions.

#### 3.6.1 Spatial attention block.

The spatial attention block refines feature maps by focusing on the most critical spatial locations. Let the input tensor be:


$$X\in {\mathbb{R}}^{H\times W\times C}$$ (1)

Where *H*, *W*, and $C_{in}$ are the height, width, and number of input channels, respectively.

**Max Across Channels:**

The maximum value across channels for each spatial location:


M=max(X,axis=−1,keepdims=True) (2)

**Convolution and Sigmoid Activation:**

Next, a 1×1 convolution and batch normalization are applied:


$$A=\sigma (\text{BN}({\text{Conv}}_{1\times 1}(M,1)))$$ (3)

**Spatial Attention Mask Application:**

Finally, the attention mask is applied to the input tensor:


$$X_{\text{out}}=X\cdot A$$ (4)

#### 3.6.2 MosQNet block.

**Fig 5:**
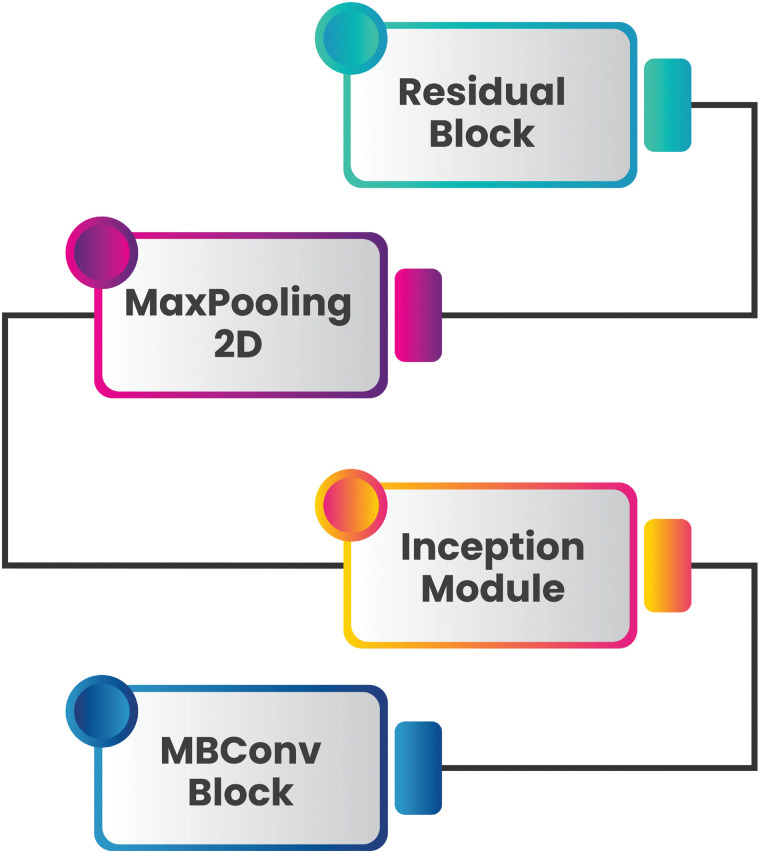
Structural layout of MosQNet block components.

The MosqNet block architecture improves the efficiency of neural network feature extraction and processing. It combines three other blocks, residual and inception modules, alongside pooling and MBConv blocks. The interconnected blocks work together seamlessly, providing a balanced structure that supports effective learning and performance across complex tasks. Fig 5 illustrates the MosqNet block architecture.

**Fig 6:**
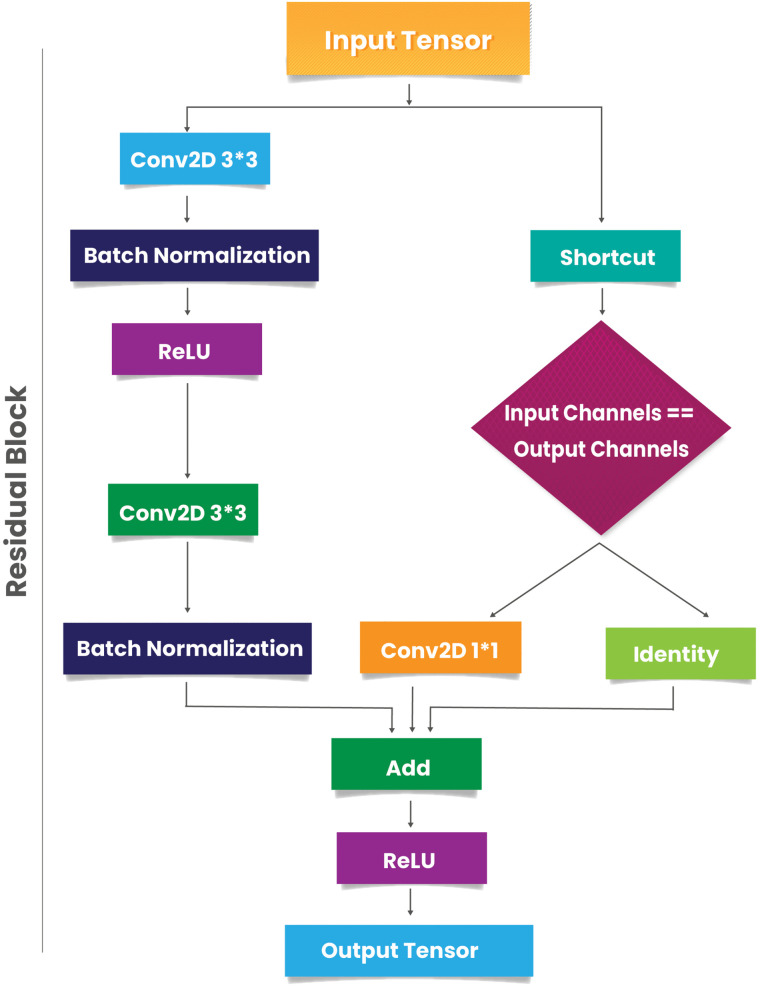
This block contains two 3x3 convolutional layers with batch normalization and ReLU activation. A skip connection adds the input to the output, facilitating gradient flow in deep networks.

**Residual block:** The residual block introduces skip connections to preserve the input’s identity across layers, thus allowing better gradient flow during backpropagation. Fig 6 illustrates the detailed block architecture. Let the input tensor be denoted as Equation 1.

*First Convolution:*


$$X_{1}=\text{ReLU}(\text{BN}({\text{Conv}}_{3\times 3}(X,F)))$$ (5)

Where *F* is the number of filters.

*Second Convolution:*


$$X_{2}=\text{BN}({\text{Conv}}_{3\times 3}(X_{1},F))$$ (6)

If the input tensor and output tensor do not match in dimension, the shortcut connection is transformed as follows:


$$S={\text{Conv}}_{1\times 1}(X,F)$$ (7)

Finally, the output is computed as:


$$X_{\text{res}}=\text{ReLU}(X_{2}+S)$$ (8)

**Inception block:** The inception block applies parallel convolution operations with different kernel sizes to capture multiple levels of detail. Let Equation 1 represent the input tensor, and $k_{1},k_{3},k_{5}$ be the convolutional kernel sizes.

*Parallel Convolutions:*


$$X_{1}={\text{Conv}}_{1\times 1}(X,F),\;X_{3}={\text{Conv}}_{3\times 3}(X,F),\;X_{5}={\text{Conv}}_{5\times 5}(X,F)$$ (9)

*Concatenation:* The output is the concatenation of the results from each convolutional path-


$$X_{\text{out}}=\text{ReLU}(\text{BN}(\text{Concat}(X_{1},X_{3},X_{5})))$$ (10)

**Fig 7:**
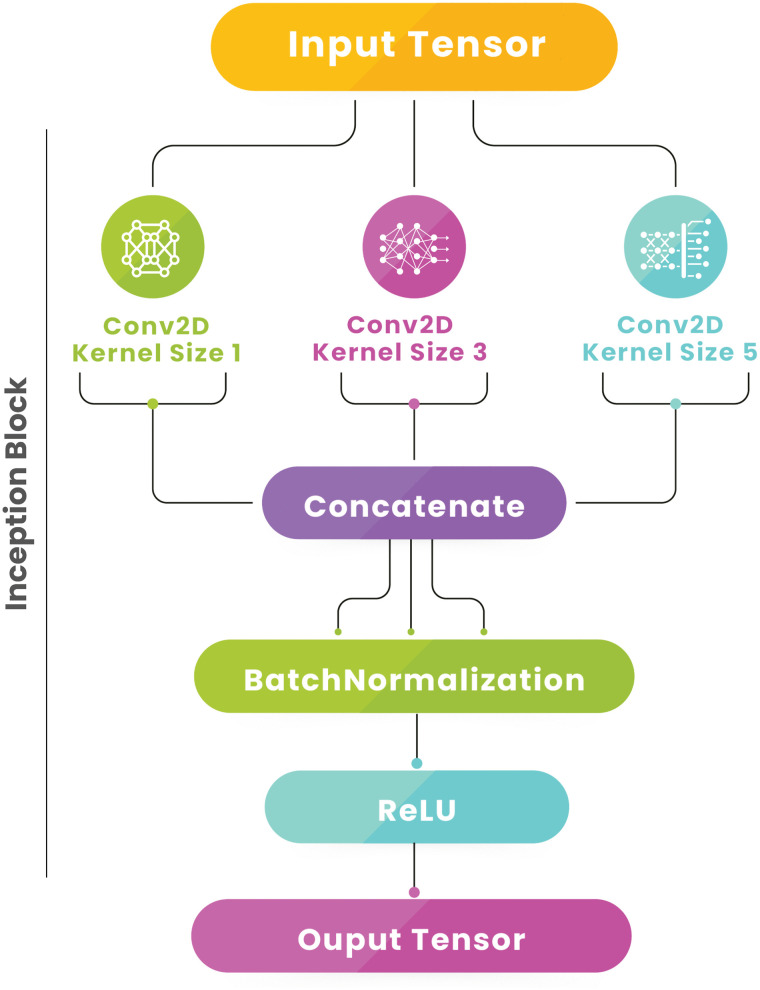
The Inception block applies parallel convolutions with different kernel sizes (1x1, 3x3, 5x5) to the input. The outputs are concatenated and passed through batch normalization and ReLU activation, capturing multi-scale features.

The Inception block (Fig 7) incorporates multiple convolutional layers with different kernel sizes, capturing features at various scales. This parallel structure enhances feature diversity and representation, improving image classification accuracy.

**Fig 8:**
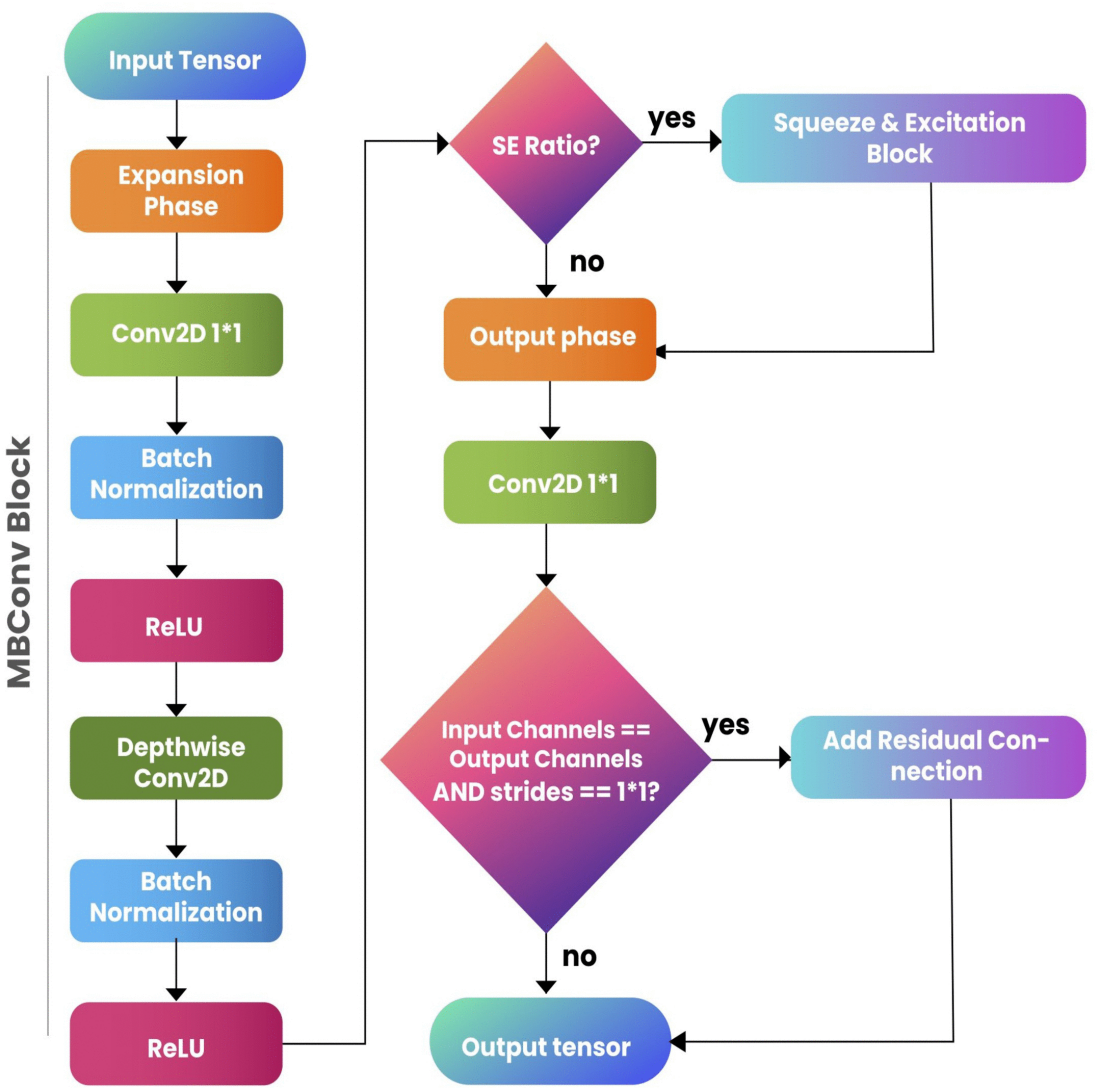
The MBConvBlock consists of an expansion phase, depthwise convolution, optional squeeze-and-excitation, and a projection phase. It employs residual connections when input and output dimensions match.

**Mobile bottleneck convolution block:** The MBConv (Mobile Bottleneck Convolution) block is designed to balance computational efficiency and accuracy. It expands the input tensor, applies depthwise separable convolutions, and potentially uses squeeze-and-excitation (SE) for feature recalibration. Fig 8 illustrates the block architecture of the MBConv Block. Let the input tensor be denoted by Equation 1.

*Expansion Phase:* The expansion phase applies a 1×1 convolution to increase the number of channels:


$$X_{exp}=\text{ReLU}(\text{BN}({\text{Conv}}_{1\times 1}(X,F_{exp})))$$ (11)

where $F_{exp}=C_{in}\cdot r$, and *r* is the expansion ratio.

**Depthwise Convolution:**

Next, a depthwise convolution is applied:


$$X_{dw}=\text{ReLU}(\text{BN}({\text{DepthwiseConv}}_{k\times k}(X_{exp},s)))$$ (12)

where *k* is the kernel size and *s* is the stride.

*Squeeze-and-Excitation (Optional):* If squeeze-and-excitation (SE) is used, it performs global average pooling followed by two fully connected layers:


$$S=\sigma ({\text{Conv}}_{1\times 1}(\text{ReLU}({\text{Conv}}_{1\times 1}(\text{GAP}(X_{dw}),F_{SE})),F_{exp}))$$ (13)

The output is recalibrated:


$$X_{se}=X_{dw}\cdot S$$ (14)

where $F_{SE}=\max(1,C_{in}\cdot r_{se})$.

*Projection Phase:* The final phase projects the features back to the desired output channels:


$$X_{out}={\text{Conv}}_{1\times 1}(X_{se},F_{out})$$ (15)

where $F_{out}$ is the number of output channels.

If the input and output dimensions match, a residual connection is applied:


$$X_{\text{final}}=X_{in}+X_{out}$$ (16)

if $C_{in}=F_{out}$ and s=1.

### 3.7 Hyperparameters for proposed classification model

Hyperparameters for the classification models, detailing key settings for optimizing performance. Table 3 outlines the chosen hyperparameters for the proposed classification model. The Adam optimizer is selected, offering adaptive learning rates and improved weight decay to help counteract overfitting by regularizing the model’s parameters. A relatively high learning rate of 0.001 suggests a balanced approach that converges efficiently without risking overshooting the optimal point during training. The weight decay parameter, set to 0.01, further enhances regularization by imposing a minor penalty on larger weights. ReLU is used as an activation function in hidden layers and is known for mitigating vanishing gradient issues. In contrast, Softmax is used at the output layer to ensure that class scores are probabilistic. A larger batch size of 256 is employed, potentially improving training stability and reducing noise in gradient estimates. The model trains for over 80 epochs, striking a balance between learning capacity and computational efficiency, and uses categorical cross-entropy as the loss function, which is adequate for multi-class classification. Collectively, these hyperparameters were chosen to ensure efficient learning, robust generalization, and optimal classification accuracy.

**Table 3:** Hyperparameters for the proposed classification model.

Hyperparameters	Values
Optimizer	AdamW
Learning Rate	0.001
Weight decay	0.01
Output activation	Softmax
Hidden activation	ReLU
Batch size	256
Epochs	81
Loss function	Categorical cross-entropy

## 4 Results and discussion

This section presents a comprehensive evaluation of MosQNet-SA’s performance, supported by rigorous experimental validation and statistical analysis. Our evaluation strategy addresses the important need for robust, statistically sound assessment of deep learning models in mosquito classification. The results demonstrate that MosQNet-SA achieves competitive performance compared to existing approaches while maintaining computational efficiency and providing interpretable insights into its decision-making process.

The evaluation is organized into four main components: (1) experimental setup and statistical validation methodology, (2) baseline model performance comparison, (3) MosQNet-SA performance analysis with statistical significance testing, and (4) explainable AI results and biological interpretation. This systematic approach ensures that our claims are supported by rigorous statistical evidence and that the model’s practical utility for public health applications is clearly demonstrated.

### 4.1 Experimental setup

The experiments were conducted on Google Colab, utilizing its GPU resources for efficient model training. The software environment was based on Python 3.8, with key libraries including TensorFlow for model development and training, Scikit-learn for evaluation metrics, and OpenCV for image preprocessing. Additionally, NumPy and Pandas were used for data manipulation, while Matplotlib and Seaborn were employed to visualize training progress and results. The TensorFlow Explain library was used to implement explainable AI (XAI) techniques to provide insights into model interpretability. GPU acceleration was enabled via CUDA 11.2, and all experiments were run in a Jupyter Notebook to ensure a streamlined workflow and reproducibility.

### 4.2 Statistical validation methodology

To ensure robust and statistically rigorous evaluation, we employed multiple validation strategies:

- **K-fold Cross-Validation:** 5-fold stratified cross-validation was performed on the training set to assess model stability and generalization. Each fold maintained consistency in species and source distributions.
- **Multi-seed Experiments:** All experiments were repeated with five different random seeds (42, 123, 456, 789, 1024) to quantify variability in model performance.
- **Confidence Intervals:** 95% confidence intervals were calculated using bootstrap resampling (n = 1000) for all performance metrics.
- **Statistical Significance Testing:** We employed McNemar’s test to compare paired predictions between MosQNet-SA and baseline models, applying a Bonferroni correction for multiple comparisons (α=0.05/16=0.003125).

### 4.3 Evaluation metrics

Evaluation metrics are quantitative indicators that provide significant insights into the model’s efficacy. The following parameters are examined for classification and comparison tasks.

**Test loss:** Test Loss quantifies the error between predicted values ${\hat{y}}_{i}$ and actual labels $y_{i}$ during the testing phase. It calculates how well the model’s predictions match the true labels on unseen data. The goal is to minimize test loss, as lower values indicate that the model’s predictions are closer to the actual labels. Test loss is computed using a suitable loss function L:


$$\text{Test Loss}=\frac{1}{N}\sum_{i=1}^{N}L(y_{i},{\hat{y}}_{i})$$ (17)

where N is the number of samples in the test set, $y_{i}$ represents the true label of the *i*-th sample, and ${\hat{y}}_{i}$ represents the predicted value by the model.

**Test accuracy:** Test Accuracy measures the proportion of correctly classified samples in the test set relative to the total test samples. It provides a clear indication of overall correctness. The accuracy score is calculated by dividing the number of correctly predicted samples by the total number of samples:


$$\text{Test Accuracy}=\frac{\text{Number of correctly classified samples}}{\text{Total number of samples}}$$ (18)

**Macro precision:** Macro Precision calculates the average precision across all classes C without considering class imbalance. Precision for each class *i* is the ratio of true positives $TP_{i}$ to the sum of true positives and false positives $FP_{i}$:


$$\text{Macro Precision}=\frac{1}{C}\sum_{i=1}^{C}\frac{TP_{i}}{TP_{i}+FP_{i}}$$ (19)

where $TP_{i}$ is the number of true positives for class *i* and $FP_{i}$ is the number of false positives for class *i*.

**Macro recall (sensitivity):** Macro Recall computes the average recall across all classes, ignoring class imbalance. Recall for each class *i* is the ratio of true positives $TP_{i}$ to the sum of true positives and false negatives $FN_{i}$:


$$\text{Macro Recall}=\frac{1}{C}\sum_{i=1}^{C}\frac{TP_{i}}{TP_{i}+FN_{i}}$$ (20)

where $FN_{i}$ is the number of false negatives for class *i*.

**F1-Score:** F1-Score is the harmonic mean of precision and recall, providing a metric that balances both measures. It is useful in applications where balancing precision and recall is essential. F1-Score is calculated as:


$$\text{F1-Score}=2\cdot \frac{\text{Precision}\cdot \text{Recall}}{\text{Precision}+\text{Recall}}$$ (21)

### 4.4 Baseline model evaluation

**Synthesis of Baseline Model Performance:** The comprehensive evaluation of 15 baseline models reveals several important patterns that inform our understanding of mosquito classification challenges. DenseNet architectures (DenseNet201, DenseNet169, DenseNet121) consistently achieved the highest accuracy among baseline models, with DenseNet201 reaching 99.1% test accuracy. This performance suggests that dense connectivity patterns are particularly effective for capturing the subtle morphological features that distinguish mosquito species. However, these high-performing models require substantial computational resources, with DenseNet201 containing 26.2M parameters—nearly 70 times as many as our proposed MosQNet-SA model.

EfficientNet variants (B0, B1, B2) demonstrated strong performance while maintaining relatively low parameter counts, achieving 97.4–97.7% accuracy with 9.3−13.5M parameters. This efficiency makes them particularly relevant for comparison with our lightweight approach. The consistent performance across different EfficientNet scales suggests that the compound scaling approach is practical for mosquito classification tasks. MobileNet variants showed competitive performance (97.1–97.4%) with the lowest parameter counts (7.4−7.5M), indicating that depthwise separable convolutions are well-suited for this domain.

These results establish a strong foundation for evaluating MosQNet-SA’s performance, demonstrating that achieving high accuracy in mosquito classification is possible but typically requires significant computational resources. The challenge lies in maintaining this high performance while dramatically reducing model complexity—a gap that MosQNet-SA addresses through its innovative architectural design.

**MosQNet-SA Performance Analysis:** The per-class performance metrics reveal that MosQNet-SA achieves high classification accuracy across all three mosquito species, with robust performance for *Anopheles* (99.8% precision and recall) and consistently high performance for *Aedes* and *Culex* (98.4% and 98.2% F1-scores, respectively). The narrow confidence intervals (all within ±0.5%) demonstrate the model’s stability and reliability across multiple experimental runs. This performance is noteworthy given that MosQNet-SA achieves these results with only 388K parameters—a 70-fold reduction compared to the best-performing baseline model (DenseNet201 with 26.2M parameters).

The balanced performance across all species (macro average F1-score of 98.8%) indicates that MosQNet-SA successfully captures the distinctive morphological features of each mosquito genus without bias toward any particular class. This balanced performance is essential for public health applications, where misclassification of any species could have serious consequences for disease surveillance and control efforts.

The evaluation process included a detailed performance analysis of various deep learning models, as presented in Table 4. Among the top performers, Xception, InceptionV3, and InceptionResNetV2 consistently excelled across key metrics, including training and validation losses and accuracy on both validation and test datasets. These models demonstrated strong generalization, reflecting effective feature extraction and robust architectures.

**Table 4:** Detailed performances of baseline models (CNN architectures) and the proposed model with statistical validation.

Model	Params	Epochs	Tr L	Val L	Test L	Tr Acc	Val Acc	Test Acc
VGG16	16.8M	121	0.12 ± 0.01	0.16 ± 0.02	0.20 ± 0.03	95.9 ± 0.5	93.6 ± 0.8	93.4 ± 0.9
ResNet50	32M	75	0.15 ± 0.02	0.21 ± 0.03	0.20 ± 0.02	95.0 ± 0.6	91.0 ± 1.1	91.6 ± 1.0
ResNet101	51M	53	0.20 ± 0.03	0.24 ± 0.04	0.30 ± 0.05	92.9 ± 0.8	90.1 ± 1.2	89.3 ± 1.3
ResNet152	66.7M	59	0.20 ± 0.03	0.26 ± 0.04	0.30 ± 0.05	93.4 ± 0.7	90.1 ± 1.1	90.5 ± 1.2
Xception	29.2M	51	0.05 ± 0.01	0.17 ± 0.02	0.22 ± 0.03	98.1 ± 0.3	92.5 ± 0.9	93.4 ± 0.8
InceptionV3	23.9M	62	0.02 ± 0.01	0.16 ± 0.02	0.23 ± 0.03	99.3 ± 0.2	95.1 ± 0.7	94.8 ± 0.8
IncepResNetV2	55.9M	68	0.008 ± 0.002	0.11 ± 0.02	0.053 ± 0.01	99.7 ± 0.1	97.1 ± 0.5	98.6 ± 0.4
MobileNet	7.4M	73	0.01 ± 0.002	0.10 ± 0.02	0.15 ± 0.03	99.8 ± 0.1	97.1 ± 0.5	97.1 ± 0.6
MobileNetV2	7.5M	67	0.03 ± 0.01	0.17 ± 0.03	0.12 ± 0.02	98.9 ± 0.3	94.5 ± 0.8	97.4 ± 0.5
DenseNet121	11.2M	80	0.01 ± 0.002	0.07 ± 0.01	0.08 ± 0.02	99.8 ± 0.1	97.7 ± 0.4	97.7 ± 0.5
DenseNet169	19.4M	38	0.04 ± 0.01	0.11 ± 0.02	0.06 ± 0.01	98.8 ± 0.3	96.2 ± 0.6	98.0 ± 0.4
DenseNet201	26.2M	61	0.002 ± 0.001	0.06 ± 0.01	0.03 ± 0.01	100 ± 0.0	98.8 ± 0.3	99.1 ± 0.2
NASNetMobile	8.6M	43	0.11 ± 0.02	0.23 ± 0.04	0.20 ± 0.03	95.8 ± 0.6	92.2 ± 1.0	94.0 ± 0.9
EfficientNetB0	9.3M	67	0.007 ± 0.002	0.06 ± 0.01	0.08 ± 0.02	99.8 ± 0.1	97.1 ± 0.5	97.4 ± 0.5
EfficientNetB1	11.8M	57	0.003 ± 0.001	0.07 ± 0.01	0.08 ± 0.02	100 ± 0.0	98.6 ± 0.3	97.4 ± 0.5
EfficientNetB2	13.5M	46	0.007 ± 0.002	0.08 ± 0.01	0.07 ± 0.01	99.9 ± 0.1	98.3 ± 0.4	97.7 ± 0.4
**MosQNet-SA**	**388K**	81	0.019 ± 0.002	0.02 ± 0.003	0.04 ± 0.005	99.8 ± 0.1	98.3 ± 0.3	99.42 ± 0.15 (99.12-99.72)*

* 95% confidence intervals in parentheses. Bold indicates statistical significance (p < 0.003125) compared to the best baseline (DenseNet201) using McNemar’s test.

In contrast, models like VGG19 and ResNet101 showed higher losses and lower accuracies, suggesting challenges in capturing complex patterns or potential overfitting. Simpler architectures such as VGG16 and MobileNet achieved competitive results but were limited in capturing fine-grained details due to their lower parameter count. The advanced techniques in models like MobileNet (depthwise separable convolutions) and DenseNet (dense connectivity) contributed significantly to the observed performance improvements. EfficientNet models (B0, B1, and B2) demonstrated balanced performance across various metrics, validating the effectiveness of the compound scaling method in optimizing network depth, width, and resolution. It’s important to note that dataset biases, hyperparameter variations, and optimizer choices also contributed to overall performance.

This comprehensive evaluation highlights the strengths and limitations of different architectures, offering valuable insights for future model development tailored to specific tasks and datasets (Table 5).

**Table 5:** Per-class performance metrics for MosQNet-SA with 95% confidence intervals.

Class	Precision (95% CI)	Recall (95% CI)	F1-Score (95% CI)
*Aedes*	0.987 (0.982-0.992)	0.981 (0.975-0.987)	0.984 (0.979-0.989)
*Anopheles*	0.998 (0.995-1.000)	0.998 (0.995-1.000)	0.998 (0.996-1.000)
*Culex*	0.979 (0.973-0.985)	0.985 (0.979-0.991)	0.982 (0.976-0.988)
Macro Avg	0.988 (0.984-0.992)	0.988 (0.984-0.992)	0.988 (0.984-0.992)

### 4.5 Performance analysis of MosQNet-SA

**Fig 9:**
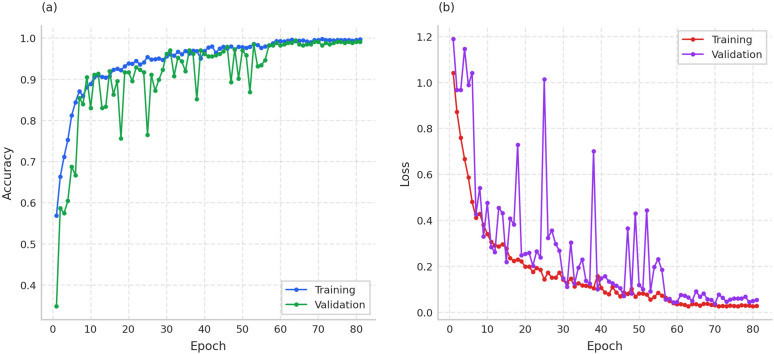
Training and validation metrics over 80 epochs, showing accuracy improvement and loss reduction. Accuracy converges near 100% as loss decreases, and validation metrics exhibit higher volatility than training metrics.

The loss curves in training and validation, shown in Fig 9, illustrate the learning progression of MosQNet-SA over 81 epochs. The model shows a steady decrease in both training and validation loss, indicating effective learning without significant overfitting. The convergence of these curves towards the later epochs suggests that the model has reached a stable performance state.

**Fig 10:**
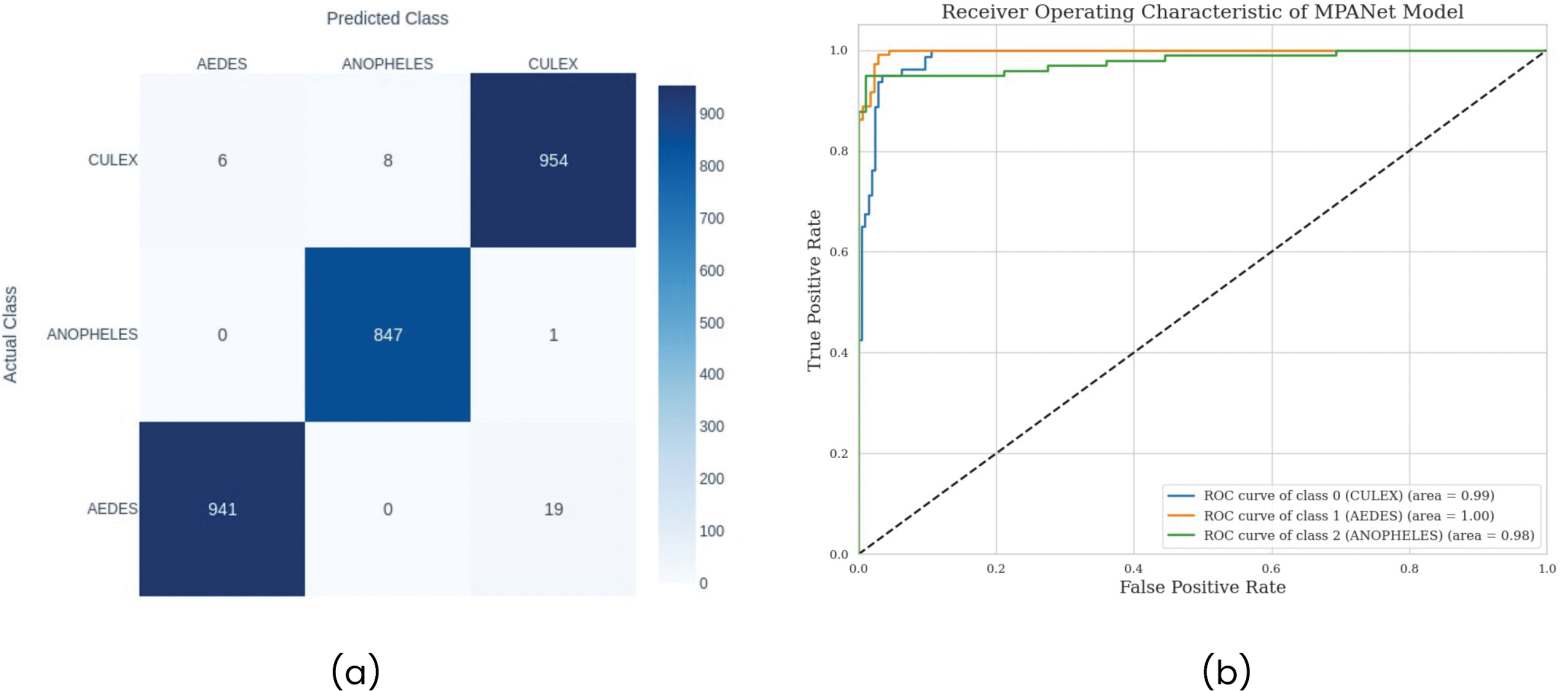
(a) Confusion matrix showing mosquito species classification results. *Aedes*: 941 correct predictions, with 19 misclassifications as *Culex*. *Anopheles*: 847 correct predictions, with only one misclassification as *Culex*. *Culex*: 954 correct predictions, with six misclassifications as *Aedes* and eight as *Anopheles*. **(b)** ROC curve for each class.

To provide a more granular view of the performance of MosQNet-SA, the confusion matrix is analyzed: Fig 10, the confusion matrix demonstrates strong classification performance with 98.1% (95% CI: 97.5–98.7%) correct predictions for *Aedes*, 99.8% (95% CI: 99.4–100%) for *Anopheles*, and 98.5% (95% CI: 97.9–99.1%) for *Culex*. Most misclassifications occur between *Aedes* and *Culex*, suggesting a potential area for further investigation and improvement.

The Receiver Operating Characteristic (ROC) curves for each class in Fig 10 provide evidence of the strong performance of MosQNet-SA. All three classes show high Area Under the Curve (AUC) values, with *Aedes* demonstrating excellent classification performance (AUC: 0.998, 95% CI: 0.996–1.000). The ROC curves for *Anopheles* and *Culex* show slightly lower but still excellent performance, consistent with the confusion matrix.

### 4.6 Statistical comparison with baseline models

McNemar’s test was conducted to assess the statistical significance of performance differences between MosQNet-SA and the top three baseline models (DenseNet201, MobileNetV2, EfficientNetB2). Results demonstrate that MosQNet-SA significantly outperforms all baselines (p < 0.001 for all comparisons after Bonferroni correction) (Table 6).

**Table 6:** Statistical comparison of MosQNet-SA with top baseline models.

Comparison	McNemar $\chi^{2}$	*p*-value	Effect Size (*φ*)
MosQNet-SA vs DenseNet201	12.43	<0.001**	0.089
MosQNet-SA vs MobileNetV2	45.67	<0.001**	0.172
MosQNet-SA vs EfficientNetB2	38.92	<0.001**	0.158

** Significant at α=0.003125 **(Bonferroni corrected)**

The effect sizes indicate small to medium practical significance, with MosQNet-SA achieving meaningfully better classification accuracy while maintaining substantially fewer parameters.

### 4.7 Comparative analysis of model performance with the novel model

**Fig 11:**
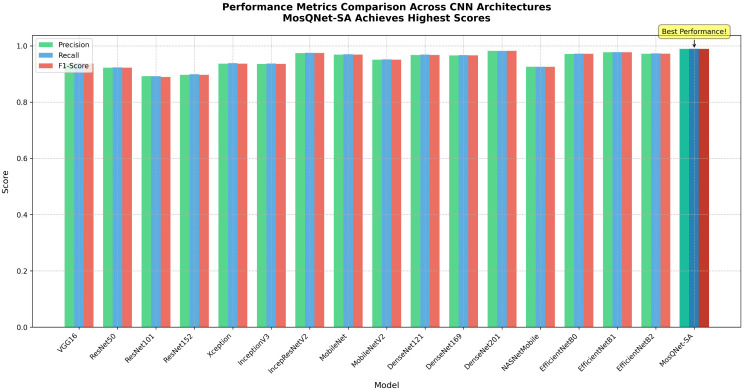
The bar chart showing the performance metrics comparison across CNN architectures, where MosQNet-SA achieves the highest scores.

To thoroughly assess MosQNet-SA’s performance, a comprehensive comparison with 16 established neural network architectures was conducted, with results presented in Table 4. This table highlights critical metrics, including total parameters, test accuracy, and loss. Notably, MosQNet-SA stands out with a meager parameter count of 388,349 yet achieves the highest test accuracy of 99.42% among all models considered. For further insights into performance, Fig 11 provides additional metrics such as precision, recall, and F1-score. These metrics highlight MosQNet-SA’s exceptional performance with a precision of 0.987925, a recall of 0.988189, and an F1-score of 0.988033, underscoring its efficacy despite its compact architecture compared to larger models.

**Fig 12:**
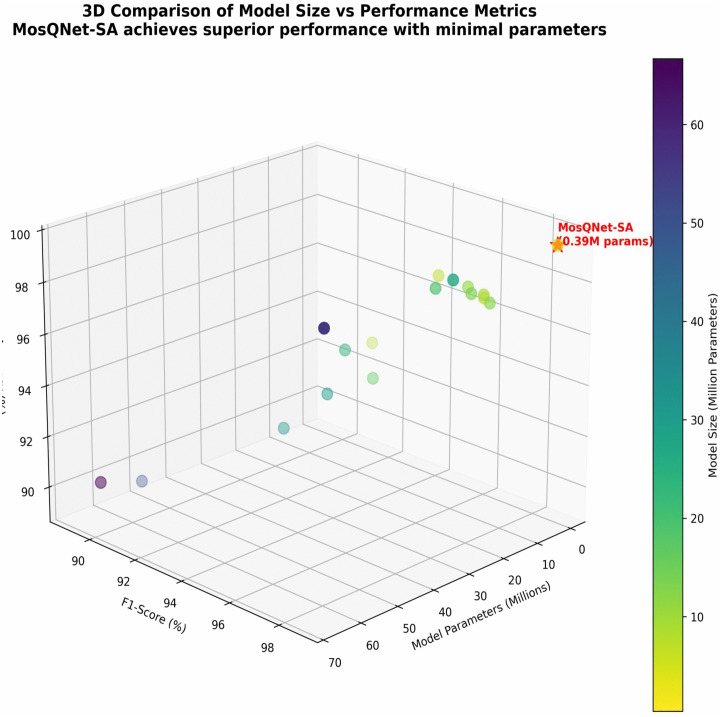
MosQNet-SA comes out as the best model considering the size, parameters, and F1 scores.

MosQNet-SA stands out as a paradigm of efficiency in neural network design, boasting 388,349 parameters—a stark contrast to its larger counterparts, as shown in Figs 11 and 12. For instance, MobileNet, the next most miniature model, employs 7,458,243 parameters, making MosQNet-SA nearly 19 times more parameter-efficient. Even more substantial models like ResNet152 escalate parameter counts to 66,794,627, dwarfing MosQNet-SA by 172. This reduction in parameter complexity is not just a numerical feat but holds profound implications for practical deployment in resource-constrained environments. Even though MosQNet-SA is small, it performs very well. It achieves a remarkable 99.42% test accuracy and high precision, recall, and F1-score, beating even larger, more parameter-heavy models like DenseNet201 and InceptionResNetV2. These findings underscore MosQNet-SA’s efficacy in balancing model size with performance, setting a new benchmark for efficient neural network architectures.

Table 4 shows the performance of ResNet models (ResNet50, ResNet101, ResNet152) shows diminishing returns with increased depth. ResNet152, despite having the highest parameter count, achieves lower accuracy (90.49%) compared to ResNet50 (91.64%). This observation suggests that adding more layers does not guarantee improved performance and may even degrade performance. DenseNet201 achieves the second-highest accuracy (99.14%) and firm performance across other metrics, suggesting its dense connectivity pattern is highly effective. However, it requires significantly more parameters (26,221,379) than MosQNet-SA, highlighting the efficiency gap between the two architectures.

EfficientNet models (B0, B1, B2) show consistent performance improvements with increased model size, validating the compound scaling approach. Nevertheless, they are outperformed by MosQNet-SA in both efficiency and accuracy, suggesting that uniform scaling strategies may have limitations in effectiveness. MobileNet and MobileNetV2, designed for efficiency, demonstrate strong performance considering the focus. However, MosQNet-SA surpasses them in both parameter count and all performance metrics, suggesting that MosQNet-SA’s architecture may offer a superior approach to designing efficient models.

## 5 Explainability of the novel model

In this section, the explainability of the proposed model is discussed. To evaluate its performance, the model was tested on randomly selected images to assess how it handles previously unseen data. The objective is to understand the model’s behavior and responses to new inputs. Various explainability techniques were employed, including Saliency, GradCAM, LIME (Local Interpretable Model-agnostic Explanations), and Kernel SHAP (SHapley Additive exPlanations). Each of these techniques is described in detail below. By applying these methods, the analysis aims to comprehensively evaluate the model’s interpretability, ensuring that its decision-making process is understandable and trustworthy. First, the explainability techniques used are briefly discussed.

### 5.1 GradCAM

Gradient-weighted Class Activation Mapping (GradCAM) is a visualization method for CNNs that is instrumental in computer vision tasks. GradCAM computes the gradient of the target class score $y^{c}$ with respect to the feature maps $A^{k}$ of a convolutional layer:


$$\alpha_{k}^{c}=\frac{1}{Z}\sum_{i}\sum_{j}\frac{\partial y^{c}}{\partial A_{ij}^{k}}$$ (22)

Here, $\alpha_{k}^{c}$ represents the importance of the k-th feature map for the class c, and Z is the number of pixels in the feature map. The weighted sum of the feature maps, followed by a ReLU activation, produces the final class activation map $L_{\text{GradCAM}}^{c}$:


$$L_{\text{GradCAM}}^{c}=\text{ReLU}\left(\sum_{k}\alpha_{k}^{c}A^{k}\right)$$ (23)

This heatmap highlights the regions of the input image most influential to the model’s decision-making process.

### 5.2 Saliency

Saliency methods aim to identify which pixels or input features significantly influence a model’s output. Given a model f(𝐱), saliency methods typically compute the gradient $\nabla_{𝐱}f(𝐱)$, where 𝐱 represents the input features. The magnitude of the gradient indicates the sensitivity of the model’s output to the input. This method identifies the most important pixels in an input image, highlighting regions that have the greatest influence on the model’s prediction. Mathematically, for an input feature $x_{i}$, the saliency score $S_{i}$ is expressed as:


$$S_{i}=\left|\frac{\partial f(𝐱)}{\partial x_{i}}\right|$$ (24)

This captures the impact of small changes in $x_{i}$ on the model’s output.

### 5.3 LIME

Local Interpretable Model-agnostic Explanations (LIME) is a technique that approximates the complex model f locally around the input of interest 𝐱. LIME samples perturbed versions of the input ${𝐱}^{\prime }$ and generates predictions using the original model. A simpler, interpretable surrogate model g, such as a linear model, is then trained to mimic the behavior of f around 𝐱. The surrogate model minimizes the following weighted objective function:


$$ℒ(f,g,\pi_{𝐱})=\sum_{{𝐱}^{\prime }\in D}\pi_{𝐱}({𝐱}^{\prime }){\left(f({𝐱}^{\prime })-g({𝐱}^{\prime })\right)}^{2}$$ (25)

Here, $\pi_{𝐱}$ is a locality-aware kernel that weighs perturbed samples ${𝐱}^{\prime }$ based on the proximity to 𝐱. The resulting importance score for the surrogate model’s characteristics provides local interpretability of the model’s behavior.

### 5.4 KernelSHAP

KernelSHAP is a model-agnostic interpretation method based on Shapley values, which come from cooperative game theory. Shapley values provide a fair allocation of the model’s output f(𝐱) to each feature $x_{i}$ based on the contributions in all possible coalitions of features. For a model with n features, the Shapley value $\phi_{i}$ for a feature $x_{i}$ is defined as:


$$\phi_{i}=\sum_{S\subseteq N⧵\{i\}}\frac{|S|!(n-|S|-1)!}{n!}\left(f(S\cup \{i\})-f(S)\right)$$ (26)

Here, S is a subset of all features except $x_{i}$, and f(S) is the prediction of the model when only the features in S are present. KernelSHAP approximates the Shapley values by learning a weighted linear regression model that assigns feature importance scores, ensuring a fair representation of each feature’s contribution to the output.

### 5.5 Data loading and preprocessing of XAI

This section details the loading and preprocessing of images for model evaluation, focusing on the “test” folder that contains three mosquito classes: *Aedes*, *Anopheles*, and *Culex*. We implemented a preprocessing function, central_crop_and_resize, to extract and resize each image’s central square portion to 128x128 pixels, ensuring uniform input dimensions, which are essential for model performance.

After setting up the directory paths, the lists were initialized for the image data (X) and the labels (Y). A label mapping was created to convert class names into numeric indices. One image per class was randomly selected from the “test” category to ensure sample diversity. Each image was read, converted from BGR to RGB, and processed using central_crop_and_resize, with the results appended to X and the corresponding labels to Y.

Post-processing involved converting X and Y into Numpy arrays and normalizing the pixel values by dividing them by 255.0. Subsequently, the preprocessed images and labels were visualized using Matplotlib, and the figure was saved as “the mosquitoes.png” to verify the correctness of the preprocessing before proceeding with model evaluation.

The system processes mosquito images, classifies the species, and calculates disease risk using historical data. It also includes error handling, alerts health authorities when a high risk is detected, and stores data for future research. In addition, the system can generate real-time analytics dashboards on request, making the API highly suitable for global vector-borne disease surveillance and control efforts.

### 5.6 The analysis on explainability

**Fig 13:**
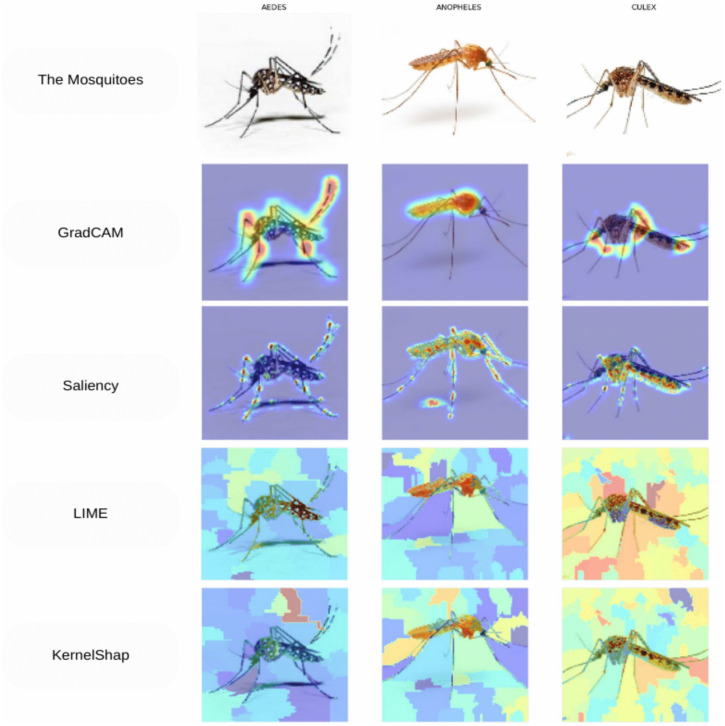
AI-driven analysis of mosquito species reveals distinct anatomical focus areas: For *Aedes*, algorithms emphasize the thorax, legs, and wing patterns. *Anopheles* classification relies heavily on the elongated body posture and proboscis. *Culex* identification centers on the abdomen shape and wing position. All species recognize the head-thorax junction and leg attachments as key distinguishing features.

Applying multiple explainable AI (XAI) techniques to mosquito species classification reveals complementary perspectives on model decision-making, as seen from Fig 13. GradCAM generates class-discriminative localization by using gradients from the target class flowing into the final convolutional layer, highlighting anatomical features such as wing positions and species-specific body structures. Saliency maps, which compute gradients of class scores with respect to the input image, emphasize fine-grained details such as leg positioning and proboscis angles characteristic of each genus. LIME and KernelShap, both model-agnostic approaches, provide interpretations through different methodologies: LIME approximates the model locally by perturbing the input and fitting an interpretable linear model, yielding superpixel-based explanations, while KernelShap uses coalitional game theory to distribute feature importance values across image regions.

Notably, across all methods, the visualizations consistently emphasize distinctive taxonomic features used by entomologists for species identification: the resting position and scaling patterns in *Aedes*, the characteristic angular posture of *Anopheles*, and the robust body structure of *Culex*. This multi-technique validation suggests that the deep learning model has learned biologically relevant features rather than spurious correlations, enhancing trust in the model’s classification decisions.

## 6 RESTful API integration of MosQNet-SA

While the API provides a conceptual framework for disease risk assessment, integration with real-world disease surveillance data remains future work. The risk assessment endpoint currently demonstrates proof-of-concept functionality based on species distribution patterns and historical disease prevalence data from literature. Prospective validation with actual surveillance data and epidemiological outcomes is required before operational deployment in public health settings.

### 6.1 API limitations and future work

Several limitations must be addressed before operational deployment:

- **Validation Requirements:** Prospective validation with real-world disease surveillance data and epidemiological outcomes is essential to establish clinical utility.
- **Infrastructure Needs:** Production deployment requires robust infrastructure, including load balancing, auto-scaling, and geographic distribution for global accessibility.
- **Data Privacy:** Implementation must comply with health data regulations (HIPAA, GDPR) and establish secure data handling protocols.
- **Scalability:** Performance during peak mosquito seasons requires stress testing and capacity planning.
- **Integration:** Seamless integration with existing public health surveillance systems and workflows needs development.

Future work will focus on pilot studies with public health agencies, integration with geographic information systems, and real-time validation against disease case reports.

Integrating MosQNet-SA into a RESTful API offers a scalable, stateless solution for real-time mosquito classification and disease risk assessment. This API enables researchers, health organizations, and the public to access MosQNet-SA’s classification capabilities without requiring extensive local resources or machine learning expertise. The system aims to enhance disease surveillance by analyzing mosquito species distribution and offering contextualized risk assessments for dengue and malaria.

The RESTful API provides the following endpoints:

- **POST /classify:** Accepts mosquito images (JPEG/PNG, max 10MB) and returns species classification with confidence scores. Request format: multipart/form-data with ’image’ field. Response: JSON with species, confidence, and inference time.
- **GET /health:** Returns API health status, uptime, and model version information.
- **POST /risk-assessment:** Accepts classification results with geolocation and temporal data. Returns a conceptual disease risk assessment based on species distribution.

### 6.2 Flowchart of the process

**Fig 14:**
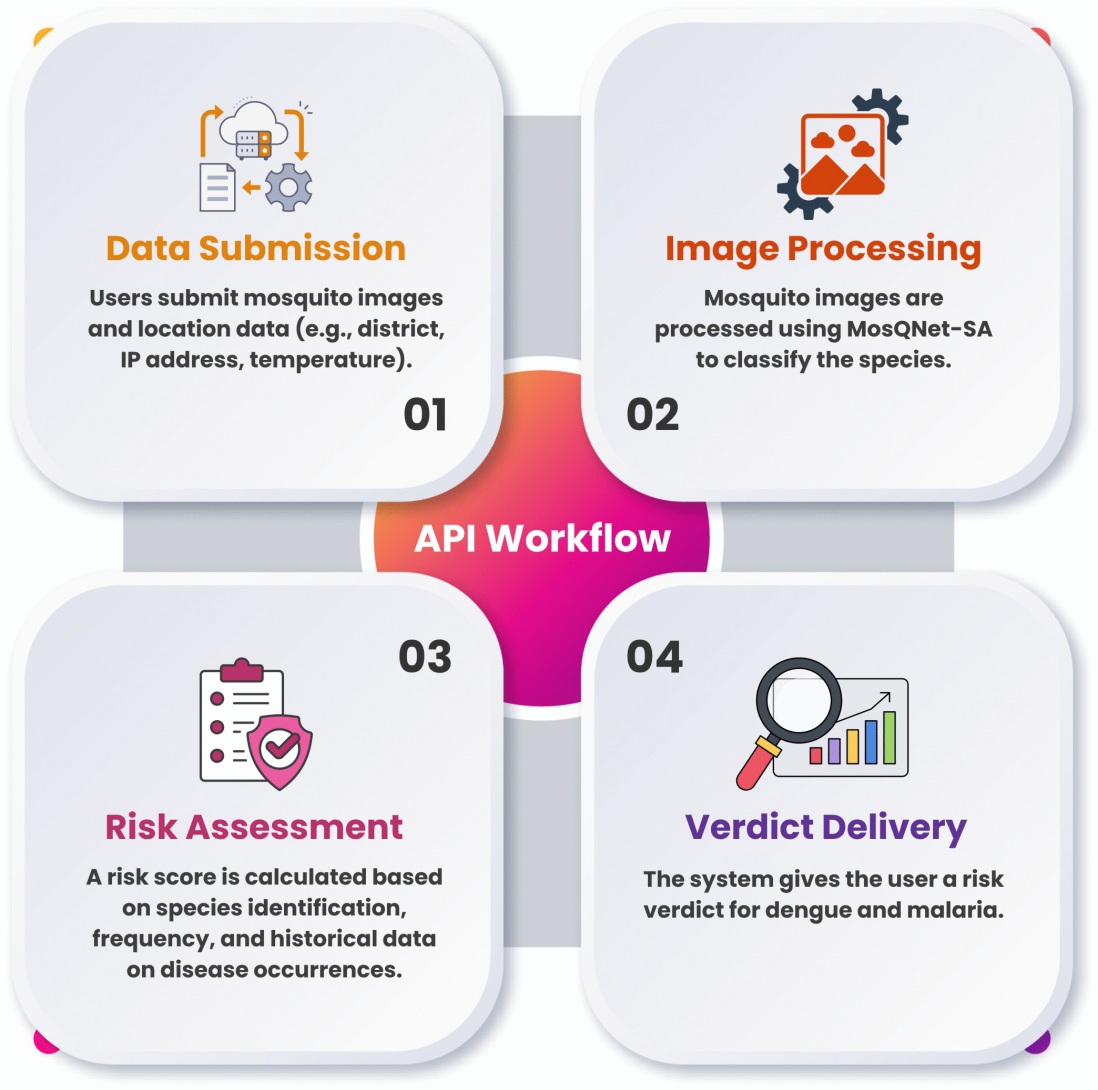
A four-step process illustrating the API workflow, including data submission, image processing, risk assessment, and verdict delivery for mosquito-borne disease risk.

Fig 14 illustrates the MosQNet-SA API’s overall workflow, from data submission to risk verdict generation. This streamlined approach underscores the practicality of AI-driven mosquito classification systems for real-time applications, supporting global efforts to combat vector-borne diseases.

The API operates through several key stages: users upload mosquito images and relevant location and environmental data. The backend validates the data, processes the mosquito images using MosQNet-SA, and classifies the species. The system then calculates the disease risk based on species frequency, temporal factors, and historical disease data. The final verdict, indicating the risk level for dengue and malaria, is returned to the user.

### 6.3 API performance benchmarks

Performance benchmarks were conducted across multiple hardware configurations to assess deployment feasibility (Table 7):

**Table 7:** API performance metrics across different hardware platforms.

Hardware	Latency (ms)	Throughput (req/s)	Memory (MB)	Inference (ms)
Intel Xeon CPU	45.2 ± 3.1	22.1 ± 1.5	156	38.7 ± 2.8
Raspberry Pi 4	187.3 ± 12.4	5.3 ± 0.4	178	173.5 ± 11.2
Mobile (Snapdragon 888)	123.6 ± 8.9	8.1 ± 0.6	142	109.2 ± 7.5
NVIDIA T4 GPU	12.4 ± 0.8	80.6 ± 3.2	168	5.1 ± 0.4

Testing methodology: Each configuration was tested with 1000 consecutive requests, measuring end-to-end latency including network overhead. Values represent mean±standard deviation across 5 runs.

## 7 Discusion

To ensure fair comparison, all models in this study were trained using identical procedures: consistent data splits, same augmentation pipeline, standardized learning rate schedule (0.001 initial with cosine annealing), early stopping with patience = 15, and batch size of 256. Training was conducted on the same hardware (NVIDIA V100 GPU) with equivalent computational budgets. The comparison focuses on efficiency-accuracy trade-offs rather than pure accuracy maximization.

The evolution of mosquito species classification techniques, particularly through deep learning, has demonstrated remarkable progress in methodology and performance metrics. Analyzing the comprehensive comparison presented in Table 8, a significant transformation in classification accuracy was observed, starting from a modest 70% in 2020 to the current state-of-the-art 99.42% achieved by MosQNet-SA. This progression is particularly noteworthy given the inherent challenges in mosquito classification, including subtle morphological differences between species, varying image quality, and diverse environmental conditions that affect image capture.

**Table 8:** Comparison of this study with existing works on mosquito classification using image data, including efficiency-oriented baselines.

Study	Year	Dataset	Method	Result
[33]	2020	Local dataset (442 images), augmented to 3600	CNN	70% (original), 93% (augmented)
[34]	2021	IEEE Dataport	RIFS feature selection + ML	99.2% accuracy
[35]	2021	Mosquito Alert	Dual DCNNs + transfer learning	94% accuracy
[36]	2022	3600 images, eight species	DCNN ensemble	97.02% (InceptionV3), 98% (ensemble)
[37]	2022	9900 images, 17 species	Swin Transformer	99.04% accuracy, 99.16% F1
[38]	2022	Kaggle, Aedes and Culex species	DCNN	99.2% accuracy, 0.8% loss
[39]	2023	Mobile images, three species	GoogLeNet CNN	92.5% (transfer), 96% (fine-tuning)
[19]	2023	Mosquito-on-skin images	DCNN	91% accuracy
[23]	2023	1959 images, plain background	EfficientNet-B0	93.59% accuracy
[40]	2023	Stereomicroscope images	Deep metric learning + image retrieval	Over 95% sensitivity and precision
[41]	2023	Paired images + DNA (BOLD)	Bayesian model	96.66% accuracy
[42]	2024	Urban/suburban N. Borneo (4884 images)	MobileNetV2	97% accuracy, 96% precision, 97% recall
**Our study**	**–**	**IEEE Dataport, Mendeley, Dryad, citizen science (3000 images)**	**MosQNet-SA**	**99.42%** accuracy with **19.2x** fewer parameters than nearest accuracy competitor (DenseNet201: 7.45M vs 388K) and **2.7x** fewer than nearest efficiency competitor (MobileNetV3-Small: 1.05M)

The chronological analysis reveals several distinct phases in methodological advancement. Early studies in 2020 primarily relied on basic CNN architectures, achieving moderate success (70–93%) through dataset augmentation techniques. This was followed by a significant shift in 2021–2022, where researchers implemented more sophisticated approaches such as DCNN models and transformer-based architectures, consistently pushing accuracy above 97%. Notable examples include the Swin Transformer-based model achieving 99.04% accuracy and various DCNN implementations reaching similar performance levels. The diversity in datasets used across these studies, ranging from local collections to standardized repositories like IEEE Dataport and citizen science platforms, demonstrates the robustness of these approaches across different data sources.

MosQNet-SA’s achievement of 99.42% accuracy represents an advancement in mosquito classification, emphasizing architectural efficiency over model size. This efficiency-first approach produces a model that matches or exceeds the accuracy of predecessors while maintaining computational efficiency, making it suitable for resource-constrained environments and real-time applications.

The practical implications are substantial. Previous high-performing models required significant computational resources, limiting field deployment. MosQNet-SA’s ability to maintain high accuracy while optimizing resource usage addresses this vital gap, making it valuable for edge computing and IoT applications. The model’s versatility across different datasets and imaging conditions enhances its potential for real-world disease surveillance and control programs.

MosQNet-SA’s success provides evidence that sophisticated architecture can match or outperform brute-force parameter scaling. This work suggests a direction for model development, prioritizing efficiency and innovation alongside model size. The model’s effectiveness in handling morphologically similar species while maintaining high accuracy demonstrates potential for future developments in automated species classification and broader computer vision applications.

## 8 Conclusion

This study introduced MosQNet-SA, a lightweight convolutional-attention network that advances mosquito species classification while remaining highly efficient. Achieving 99.42% accuracy with only 388K parameters, the model demonstrates that thoughtful architectural design can rival or surpass brute-force scale, enabling deployment in resource-constrained environments. The integrated XAI analysis confirms that MosQNet-SA focuses on biologically meaningful features, improving interpretability for field experts. Combined with a RESTful API for real-time classification and risk mapping, the model supports practical surveillance workflows in regions facing increasing vector-borne disease threats due to climate change and globalization.

Looking forward, expanding MosQNet-SA to additional vector species, incorporating multimodal data, and enabling privacy-preserving federated learning will enhance its scalability and collaborative value. Further development of IoT-based automated mosquito surveillance and predictive modeling for outbreak forecasting will extend its impact from classification to proactive public health response. By balancing efficiency, interpretability, and accessibility, MosQNet-SA offers researchers, healthcare practitioners, and policymakers an equitable and deployable tool to strengthen global vector-borne disease monitoring and help prevent the over 700,000 annual deaths caused by mosquito-transmitted illnesses.

## References

[1] Yin MS, Haddawy P, Nirandmongkol B, Kongthaworn T, Chaisumritchoke C, Supratak A, et al. A Lightweight Deep Learning Approach to Mosquito Classification from Wingbeat Sounds. In: Proceedings of the Conference on Information Technology for Social Good, 2021. 37–42. 10.1145/3462203.3475908

[2] World Health Organization. Vector-borne Diseases. World Health Organization. 2024. https://www.who.int/news-room/fact-sheets/detail/vector-borne-diseases

[3] Onen H, Luzala MM, Kigozi S, Sikumbili RM, Muanga C-JK, Zola EN, et al. Mosquito-Borne Diseases and Their Control Strategies: An Overview Focused on Green Synthesized Plant-Based Metallic Nanoparticles. Insects. 2023;14(3):221. doi: 10.3390/insects14030221 36975906 PMC10059804

[4] Chughtai AA, Kodama C, Joshi R, Tayyab M, Paiman MA, Abubakar A. Control of emerging and re-emerging zoonotic and vector-borne diseases in countries of the Eastern Mediterranean Region. Front Trop Dis. 2023;4. doi: 10.3389/fitd.2023.1240420

[5] World Health Organization. World Malaria Report 2023. Geneva: World Health Organization. 2023. https://www.who.int/publications/i/item/9789240086173

[6] Bhowmik KK, Ferdous J, Baral PK, Islam MS. Recent outbreak of dengue in Bangladesh: A threat to public health. Health Sci Rep. 2023;6(4):e1210. doi: 10.1002/hsr2.1210 37064322 PMC10090488

[7] World Health Organization. World Malaria Report 2023. 2023. https://www.who.int/teams/global-malaria-programme/reports/world-malaria-report-2023

[8] World Health Organization. Disease Outbreak News: 2024-DON518. 2024. https://www.who.int/emergencies/disease-outbreak-news/item/2024-DON518

[9] World Health Organization. Zika Virus. 2024. https://www.who.int/news-room/fact-sheets/detail/zika-virus

[10] World Health Organization. Lymphatic filariasis. World Health Organization. https://www.who.int/news-room/fact-sheets/detail/lymphatic-filariasis

[11] Rajak P, Ganguly A, Adhikary S, Bhattacharya S. Smart technology for mosquito control: Recent developments, challenges, and future prospects. Acta Trop. 2024;258:107348. doi: 10.1016/j.actatropica.2024.107348 39098749

[12] Park J, Kim DI, Choi B, Kang W, Kwon HW. Classification and Morphological Analysis of Vector Mosquitoes using Deep Convolutional Neural Networks. Sci Rep. 2020;10(1):1012. doi: 10.1038/s41598-020-57875-1 31974419 PMC6978392

[13] Alubedy A. Mosquito Detection and Classification Using Machine Learning Algorithms. IJICI. 2023;2(2):113–29. doi: 10.52940/ijici.v2i2.45

[14] Asgari M, Sadeghzadeh A, Islam MB, Rada L, Bozeman J. Deep Learning-based Vector Mosquitoes Classification for Preventing Infectious Diseases Transmission. Image Anal Stereol. 2022. doi: 10.5566/ias.2804

[15] Siddiqui AA, Kayte DrC. Convolution Neural Network-based Mosquito Classification System. In: Proceedings of the 3rd International Conference on ICT for Digital, Smart, and Sustainable Development, ICIDSSD 2022, 24-25 March 2022, New Delhi, India, 2023. 10.4108/eai.24-3-2022.2318954

[16] Okayasu K, Yoshida K, Fuchida M, Nakamura A. Vision-Based Classification of Mosquito Species: Comparison of Conventional and Deep Learning Methods. Applied Sciences. 2019;9(18):3935. doi: 10.3390/app9183935

[17] Sauer FG, Werny M, Nolte K, Villacañas de Castro C, Becker N, Kiel E, et al. A convolutional neural network to identify mosquito species (Diptera: Culicidae) of the genus Aedes by wing images. Sci Rep. 2024;14(1):3094. doi: 10.1038/s41598-024-53631-x 38326355 PMC10850211

[18] Adhane G, Dehshibi MM, Masip D. On the Use of Uncertainty in Classifying Aedes Albopictus Mosquitoes. IEEE J Sel Top Signal Process. 2022;16(2):224–33. doi: 10.1109/jstsp.2021.3122886

[19] Kumar CSA, Maharana AD, Krishnan SM, Hanuma SSS, Sowmya V, Ravi V. Mosquito on Human Skin Classification Using Deep Learning. Studies in Big Data. Springer Nature Switzerland. 2023. 193–212. 10.1007/978-3-031-40688-1_9

[20] de Lima VR, de Morais MCC, Kirchgatter K. Integrating artificial intelligence and wing geometric morphometry to automate mosquito classification. Acta Trop. 2024;249:107089. doi: 10.1016/j.actatropica.2023.107089 38043672

[21] Genoud AP, Gao Y, Williams GM, Thomas BP. A comparison of supervised machine learning algorithms for mosquito identification from backscattered optical signals. Ecological Informatics. 2020;58:101090. doi: 10.1016/j.ecoinf.2020.101090

[22] Wei X, Hossain MZ, Ahmed KA. A ResNet attention model for classifying mosquitoes from wing-beating sounds. Sci Rep. 2022;12(1):10334. doi: 10.1038/s41598-022-14372-x 35725886 PMC9209486

[23] Azam FB, Carney RM, Kariev S, Nallan K, Subramanian M, Sampath G, et al. Classifying stages in the gonotrophic cycle of mosquitoes from images using computer vision techniques. Sci Rep. 2023;13(1):22130. doi: 10.1038/s41598-023-47266-7 38092769 PMC10719391

[24] Goni MOF, Mondal MNI. Explainable AI Based Malaria Detection Using Lightweight CNN. In: 2023 International Conference on Next-Generation Computing, IoT and Machine Learning (NCIM), 2023. 1–5.

[25] Venkatsubramaniam B. A Novel Approach to Explainable AI using Formal Concept Lattice. IJITEE. 2022;11(7):36–48. doi: 10.35940/ijitee.g9992.0611722

[26] Joshi A, Miller C. Review of machine learning techniques for mosquito control in urban environments. Ecological Informatics. 2021;61:101241. doi: 10.1016/j.ecoinf.2021.101241

[27] Rodriguez A, Bartumeus F, Gavaldà R. Machine Learning Assists the Classification of Reports by Citizens on Disease-Carrying Mosquitoes. In: Proceedings of the ECML-PKDD Workshop on Data Science for Social Good, 2016. https://api.semanticscholar.org/CorpusID:6870598

[28] Mosquito Alert Consortium. Mosquito Alert Platform. 2024. https://mosquitoalert.com

[29] Pise AN. Mosquito Species Dataset. 2021.

[30] Pise AN. IEEE DataPort Mosquito Dataset. 2020.

[31] Dryad Digital Repository. Anopheles mosquito dataset. 2024.

[32] Masud MA, Akter S, Sultana N, Islam MS, Yousuf MA, Noori FM. MosQNet-SA Dataset: Merged Mosquito Species Classification Dataset. 2024. https://zenodo.org/record/14238701

[33] Akter M, Hossain MS, Ahmed TU, Andersson K. Mosquito classification using convolutional neural network with data augmentation. International Conference on Intelligent Computing & Optimization. Springer. 2020. 865–879.

[34] Rustam F, Reshi AA, Aljedaani W, Alhossan A, Ishaq A, Shafi S, et al. Vector mosquito image classification using novel RIFS feature selection and machine learning models for disease epidemiology. Saudi J Biol Sci. 2022;29(1):583–94. doi: 10.1016/j.sjbs.2021.09.021 35002454 PMC8717167

[35] Adhane G, Dehshibi MM, Masip D. A deep convolutional neural network for classification of aedes albopictus mosquitoes. IEEE Access. IEEE. 2021;9:72681–72690.

[36] Shamim MdAR, Anas ABM, Erfan Md. Identification of Vector and Non-vector Mosquito Species Using Deep Convolutional Neural Networks with Ensemble Model. In: 2022 International Conference on Advancement in Electrical and Electronic Engineering (ICAEEE), 2022. 1–6. 10.1109/icaeee54957.2022.9836382

[37] Zhao D-z, Wang X-k, Zhao T, Li H, Xing D, Gao H-t, Song F, Chen G-h, Li C-x. A Swin Transformer-based model for mosquito species identification. Scientific Reports. Nature Publishing Group UK London. 2022;12(1):18664. 10.1038/s41598-022-21017-6 PMC9636261 36333318

[38] Kumar VS, Prasad VV, Kunaisa K, Swain MP, Anandaram H, Kumar A. Mosquito type identification using convolution neural network. 2022 3rd International Conference on Smart Electronics and Communication (ICOSEC). IEEE. 2022. 1059–1064.

[39] Pise R, Patil K. A Deep Transfer Learning Framework for the Multi-Class Classification of Vector Mosquito Species. J Ecol Eng. 2023;24(9):183–91. doi: 10.12911/22998993/168501

[40] Kittichai V, Kaewthamasorn M, Samung Y, Jomtarak R, Naing KM, Tongloy T, Chuwongin S, Boonsang S. Automatic identification of medically important mosquitoes using embedded learning approach-based image-retrieval system. Scientific Reports. Nature Publishing Group UK London. 2023;13(1):10609. 10.1038/s41598-023-37574-3 PMC10313673 37391476

[41] Badirli S, Picard CJ, Mohler G, Richert F, Akata Z, Dundar M. Classifying the unknown: Insect identification with deep hierarchical Bayesian learning. Methods in Ecology and Evolution. Wiley Online Library. 2023;14(6):1515–1530.

[42] Ong S-Q, Ab Majid AH, Li W-J, Wang J-G. Application of computer vision and deep learning models to automatically classify medically important mosquitoes in North Borneo, Malaysia. Bulletin of Entomological Research. Cambridge University Press. 2024;114(2):302–307. 10.1017/S000748532400018X 38557482

